# Aura and osmophobia are associated with the *IL1A* -889C > T (rs1800587) variant in migraine

**DOI:** 10.1055/s-0044-1791200

**Published:** 2024-11-06

**Authors:** Amanda Brant Rocha, Giovana Ortiz Zendrini, Maria Paula Bertoletti Juliani, Regina Célia Poli Frederico, Valéria Aparecida Bello, Carlos Eduardo Coral de Oliveira, Edna Maria Vissoci Reiche, Aline Vitali-Silva

**Affiliations:** 1 Pontifícia Universidade Católica do Paraná, Faculdade de Medicina, Departamento de Medicina, Londrina PR, Brazil.

**Keywords:** Headache, Migraine Disorders, Interleukin-1alpha, Genetic Variation, Cefaleia, Transtornos de Enxaqueca, Interleucina-1alfa, Variação Genética

## Abstract

**Background** Migraine belongs to the group of primary headaches, affecting 14.4% of the global population. The pathophysiological mechanisms of migraine involve the interplay between hypothalamic activation, cortical spreading depression, trigeminal stimulation, and inflammatory components with neurogenic inflammation or neuroinflammation.

**Objective** To assess the frequency of the *IL1A* -899C > T (rs1800587) genetic variant in patients with migraine and healthy controls, as well as its association with the clinical manifestations of migraine.

**Methods** We conducted a case-control study involving 92 migraine patients and 88 healthy controls matched for age, sex, body mass index (BMI), and ethnicity. Demographic, anthropometric, and clinical data were obtained. The *IL1A* -889C > T (rs1800587) variant was identified using real-time polymerase chain reaction.

**Results** The study comprised predominantly women and Caucasian individuals, with no significant differences in terms of age, sex, ethnicity, or BMI observed between the migraine and control groups. Within the migraine group, 57.6% had episodic migraines, and 45.7% experienced aura. The patients carrying the CT genotype showed stronger associations with the presence of aura (CT: 57.7%; TT: 27.5%; *p* = 0.027), and those carrying the CT and TT genotypes showed higher osmophobia rates when compared with the CC genotype ( *p* = 0.003). The *IL1A* -889C > T genetic variant was not associated with migraine susceptibility, be it chronic or episodic, nor to other symptoms associated with migraine.

**Conclusion** The *IL1A* -889C > T genetic variant was associated with aura and osmophobia in migraine patients.

## Resumo

**Antecedentes** A migrânea é uma cefaleia primária que afeta 14,4% da população global. Os mecanismos fisiopatológicos da migrânea envolvem a interação entre a ativação hipotalâmica, depressão cortical alastrante, estimulação trigeminal e componentes inflamatórios com inflamação neurogênica ou neuroinflamação.

**Objetivo** Avaliar a frequência da variante genética *IL1A* -899C > T (rs1800587) em pacientes com migrânea e controles saudáveis, bem como sua associação com as manifestações clínicas da migrânea.

**Métodos** Realizamos um estudo do tipo caso-controle com 92 pacientes com migrânea e 88 indivíduos saudáveis pareados por idade, sexo, índice de massa corporal (IMC) e etnia. Dados demográficos, antropométricos e clínicos foram obtidos. A variante *IL1A* -889C > T (rs1800587) foi identificada por meio de reação em cadeia da polimerase em tempo real.

**Resultados** O estudo foi composto predominantemente por mulheres e indivíduos caucasianos, sem diferenças significativas em termos de idade, sexo, etnia e IMC entre os grupos de migrânea e controle. No grupo de migrânea, 57,6% apresentavam migrânea episódica e 45,7% tinham aura. Encontrou-se uma associação entre os pacientes portadores do genótipo CT e a presença de aura (CT: 57,7%; TT: 27,5%; *p* = 0,027), e os portadores dos genótipos CT e TT apresentaram taxas mais altas de osmofobia quando comparados com os portadores do genótipo CC ( *p* = 0,003). A variante *IL1A* -889C > T não esteve associada à suscetibilidade à migrânea, a sua forma crônica ou episódica, bem como a outros sintomas relacionados à migrânea.

**Conclusão** O polimorfismo genético *IL1A* -889C > T foi associado com aura e osmofobia em indivíduos com migrânea.

## INTRODUCTION

Migraine belongs to the group of primary headaches, and it affects 14.4% of the global population. It primarily impacts female individuals, and it constitutes a significant cause of disability in this group. 1 Migraine is also a genetic disease, with a hereditary character, which is distinct between migraine with and without aura. Compared to the general population, the first-degree relatives of individuals with migraine without aura are 1.9 times more likely to develop the disease, while the first-degree relatives of individuals with migraine with aura are approximately 4 times more likely to develop the condition. 2 There is a strong association between migraine and genetic factors. When combined with environmental factors, they are capable of determining the level of intensity, pattern of involvement, and general clinical manifestations of the patient. 3

The pathophysiological mechanisms of migraine involve the interplay between genetic and environmental factors, resulting in hypothalamic activation, cortical spreading depression, and trigeminal activation. 4 Throughout these processes, inflammatory components interact with the nervous system, either through neurogenic inflammation or neuroinflammation within the central nervous system or the trigeminal ganglion. 5

Several cytokines, including tumor necrosis factor alpha (TNF-α), interleukin-1 beta (IL-1β) and interleukin-6 (IL-6), have been associated with the pathophysiology of migraine, as increased values of these cytokines have been observed in individuals with this condition. 6 7 8 9 Interleukin-1 alpha (IL-1α) is a proinflammatory cytokine that exists constitutively as a precursor in various healthy tissues. It can be released by damaged cells into the extracellular space or expressed as a protein bound to the cell membrane, triggering a local inflammatory response. 10 11 In the brain, IL-1α is expressed by astrocytes; however, it has been relatively understudied in migraine. Boćkowski et al. 12 identified a connection between the presence of aura and higher levels of IL-1α in children and adolescents with migraine.

The biological levels of IL-1α can be influenced by genetic determinants, such as the -889 C > T (rs1800587) variant of the *IL1A* gene, which involves the substitution of cytosine by thymine in the regulatory region of said gene. This alteration can lead to interindividual variations in cytokine production levels, potentially influencing the susceptibility and clinical characteristics of migraine. 13 Rainero et al. 13 observed that carriers of the *IL1A* -889TT genotype had a lower average age at migraine onset and a higher prevalence of aura compared with the CC and CT genotypes. Conversely, Rubino et al. 14 did not find an association between the *IL1A* -889C > T variant and the response to non-steroidal anti-inflammatory drugs and triptans.

The *IL1A* -889C > T variant has been primarily investigated in periodontal disease 15 and autoimmune conditions. 16 However, data regarding its association with migraine are limited. Therefore, the present study aims to assess the frequency of the -889 C > T (rs1800587) genetic variant in the *IL1A* gene in patients with migraine and healthy controls, as well as its association with the clinical manifestations of migraine.

## METHODS

### Design and sample

The present was a case-control and association study involving 92 migraine patients and 88 healthy controls aged 18 to 60 years, of both genders, who attended the Academic Outpatient Clinic of Pontifícia Universidade Católica do Paraná (PUCPR), in the municipality of Londrina, state of Paraná, Brazil, from December 2018 to July 2021. The migraine and control groups were from the same geographic region and were matched for age, sex, body mass index (BMI), and ethnicity. Individuals with severe and uncontrolled neurological, psychiatric, or inflammatory diseases, as well as pregnant and lactating women, were excluded. The diagnosis of migraine was established according to the guidelines of the third edition of the International Classification of Headache Disorders (ICHD-III). 17 The individuals in the control group were recruited from the outpatient waiting room during routine consultations. They had no history of migraine and tested negative for migraine according to the Identification of Migraine (ID-Migraine) score. 18

The participants underwent a structured interview, providing demographic, anthropometric, and clinical data. In the case group, migraine was classified as with or without aura and as episodic or chronic, following the ICHD-III criteria. 17 Information regarding associated migraine symptoms such as phonophobia, photophobia, osmophobia, and allodynia was recorded, along with prodromal and postdromal symptoms.

### Genotyping

For the genotyping analysis, blood collection was performed via peripheral venous puncture into tubes containing ethylenediaminetetraacetic acid (EDTA) 0.6% as an anticoagulant. Genomic DNA was extracted from the buffy coat of peripheral blood cells using a resin column. The DNA samples were stored at -20° C until use. Quality and quantity of the DNA were assessed by absorbance analysis using a spectrophotometer (NanoDrop 2000 Thermo Fisher Scientific, Waltham, MA, United States) at 260 nm and 280 nm. Subsequently, DNA dilution was performed in ultrapure water to obtain a final concentration of 30 ng/μL.

The *IL1A* -889C > T (rs1800587) genetic variant was analyzed using the real-time polymerase chain reaction amplification technique with the TaqMan system (Applied Biosystems, Waltham, MA, United States). The reaction consisted of a final volume of 10 μL, including 5.25 μL of TaqMan Genotyping Master Mix (1x) (Applied Biosystems), 0.5 μL of probe (1x), 3.25 μL of ultrapure Milli-Q water (Sigma-Aldrich, Burlington, MA, United States), and 1μL of DNA (30 ng/μL). Cycling involved an initial denaturation step at 95°C for 10 minutes, followed by 50 cycles of denaturation at 95° C for 15 seconds and annealing/extension of primers at 60°C for 1 minute and 30 seconds, and a final cycle of 30 seconds at 60°C using the QuantStudio 3 thermocycler (Applied Biosystems). The QuantStudio 3 software (Applied Biosystems) was employed for single nucleotide polymorphism (SNP) allele discrimination.

### Statistical analysis

The categorical variables were expressed as absolute numbers (n) and percentages (%), and they were analyzed using the Fisher exact test or the Chi-squared test, followed by the Bonferroni post-hoc test, as appropriate. Continuous data were expressed as mean ± standard deviation (SD) values, and they were assessed using the Student *t* -test after Kolmogorov-Smirnov tests confirmed parametric data distribution. A 95% confidence interval (95%CI) and a significance level of 5% ( *p* < 0.05) were adopted. The statistical analysis was conducted using IBM SPSS Statistics for Windows (IBM Corp., Armonk, NY, United States) software, version 25.0.

### Ethical Considerations

The present study was conducted after approval by the Ethics in Research Committee of PUCPR, under review number 3.029.972, and the participants signed an informed consent form, in accordance with resolution n° 466/2012 of the Brazilian National Health Council (Conselho Nacionalo de Saúde, CNS, in Portuguese).

## RESULTS

Table 1 shows the main characteristics of the study participants. The sample consisted predominantly of women and Caucasian individuals, with mean ages of 35.48 and 36.73 years in the migraine and control groups respectively. There were no statistically significant differences in terms of age, sex, ethnicity, BMI, hypertension, or type-2 diabetes mellitus between the migraine and control groups. Among the migraine group, 53 (57.6%) individuals had the episodic form, and 42 (45.7%) presented aura. Data on phonophobia, photophobia, osmophobia, allodynia, prodrome, and postdrome are presented in Table 1 . In this sample, there were 3 patients with incomplete data on the presence of osmophobia and 1 patient with incomplete data on allodynia, prodrome, and postdrome.

**Table 1:** Clinical features of migraine patients and controls

		Migraine patients (n = 92)	Healthy controls (n = 88)	*p* -value
Age (years): mean ± SD		35.48 ± 12.91	36.73 ± 15.15	0.552
Sex: n (%)	Male	17 (18.5)	22 (25.0)	0.366
	Female	75 (81.5)	66 (75.0)	
Ethnicity: n (%)	Caucasian	69 (75.8)	57 (78.1)	0.853
	Non-Caucasian	22 (24.2)	16 (21.9)	
BMI (Kg/m ^2^ ): mean ± SD		26.49 ± 5.02	26.42 ± 6.52	0.939
SAH: n (%)	Yes	12 (13.3)	13 (14.8)	0.832
	No	78 (86.7)	75 (85.2)	
T2DM: n (%)	Yes	7 (7.8)	7 (8.0)	> 0.999
	No	83 (92.2)	81 (92.0)	
Migraine classification: n (%)	Episodic	53 (57.6)		
	Chronic	36 (42.4)		
Aura: n (%)	Yes	42 (45.7)		
	No	50 (54.3)		
Photophobia: n (%)	Yes	83 (90.2)		
	No	9 (9.8)		
Phonophobia: n (%)	Yes	72 (78.3)		
	No	20 (21.7)		
Osmophobia: n (%)	Yes	57 (62.0)		
	No	32 (32.8)		
Allodynia: n (%)	Yes	42 (45.7)		
	No	49 (53.3)		
Prodrome: n (%)	Yes	68 (73.9)		
	No	23 (25.0)		
Postdrome: n (%)	Yes	81 (88.0)		
	No	10 (10.9)		

Abbreviations: BMI, body mass index; SAH, systemic arterial hypertension; SD, standard deviation; T2DM, type-2 diabetes mellitus.

Table 2 presents the results of susceptibility analyses for migraine in the allelic, codominant, dominant, and recessive genetic models, in which no statistically significant differences were observed.

**Table 2:** Frequency of the *IL1A* -889C > T (rs1800587) variant in individuals with migraine and controls according to different genetic models

Genetic model		Migraine patients: n (%)	Healthy controls: n (%)	*p* -value
Allelic	C	74 (40.0)	81 (46.0)	0.288
	T	110 (60.0)	95 (54.0)	
Codominant	CC	11 (12.0)	14 (15.9)	0.458
	CT	52 (56.5)	53 (60.2)	
	TT	29 (31.5)	21 (26.9)	
Dominant	CT + TT	81 (88.0)	74 (84.0)	0.521
	CC	11 (12.0)	14 (16.0)	
Recessive	CC + CT	63 (68.0)	67 (76.0)	0.318
	TT	29 (32.0)	21 (24.0)	

Table 3 shows the associations between the clinical characteristics of migraine across different *IL1A* -889C > T (rs1800587) genotypes. We observed that the CT genotype was more associated with the presence of aura compared with the TT genotype (CT: 30/52 [57.7%]; TT: 8/29 [27.5%]; *p* = 0.027). Additionally, osmophobia was more frequent in the patients carrying the CT (35/51; 68.6%) and TT (20/27; 74.1%) genotypes compared carriers of the CC genotype (2/11; 18.2%) ( *p* = 0.003). Moreover, the prevalence of prodrome was higher in the CT heterozygous patients (44/52; 84.6%) than in the TT homozygotes patients (17/28; 20.7%) ( *p* = 0.042). Finally, there were no statistically significant associations involving the genetic variant and chronic or episodic migraines, nor with other symptoms associated with migraine.

**Table 3:** Associations involving the clinical characteristics of patients with migraine and the *IL1A* -889C > T (rs1800587) variant in codominant, dominant, and recessive genetic models

		Codominant				Dominant			Recessive		
		CC: n (%)	CT: n (%)	TT: n (%)	*p* -value	CC: n (%)	CT + TT: n (%)	*p* -value	CC + CT: n (%)	TT: n (%)	*p* -value
Classification	Episodic	7 (64.6)	27 (51.9)	18 (64.3)	0.654	7 (63.6)	45 (56.3)	0.752	34 (54.0)	18 (64.3	0.359
	Chronic	4 (36.4)	25 (48.1)	10 (35.7)		4 (36.4)	35 (43.8)		29 (46.0)	10 (35.7)	
Aura	Yes	4 (36.4) ^a,b^	30 (57.7) ^b^	8 (27.6) ^a^	0.027	4 (36.4)	38 (46.9)	0.510	34 (54.0)	8 (27.6)	**0.018**
	No	7 (63.6) ^a,b^	22 (42.3) ^b^	21 (72.4) ^a^		7 (63.6)	43 (53.1)		(46.0)	21 (72.4)	
Allodynia	Yes	7 (63.6)	25 (48.1)	10 (35.7)	0.265	7 (63.6)	35 (43.8)	0.215	32 (50.8)	10 (35.7)	0.183
	No	4 (36.4)	27 (51.9)	18 (64.3)		4 (36.4)	45 (56.4)		31 (49.2)	18 (64.3)	
Osmophobia	Yes	2 (18.2) ^a^	35 (68.6) ^b^	20 (74.1) ^b^	0.003	2 (18.2)	55 (70.5)	0.001	37 (59.7)	20 (74.1)	0.193
	No	9 (81.8) ^a^	16 (31.4) ^b^	7 (25.9) ^b^		9 (81.9)	23 (29.5)		25 (40.3)	7 (25.9)	
Phonophobia	Yes	9 (81.8)	41 (78.8)	22 (75.9)	0.909	9 (81.8)	63 (77.8)	> 0.999	50 (79.4)	22 (75.9)	0.705
	No	2 (18.2)	11 (21.2)	7 (24.1)		2 (18.2)	18 (22.2)		13 (20.6)	7 (24.1)	
Photophobia	Yes	10 (90.9)	47 (90.4)	26 (89.7)	0.991	10 (90.9)	73 (90.1)	> 0.999	57 (90.5)	26 (89.7)	> 0.999
	No	1 (9.1)	5 (9.6)	3 (10.3)		1 (9.1)	8 (9.9)		6 (9.5)	3 (10.3)	
Prodrome	Yes	7 (63.6) ^a,b^	44 (84.6) ^b^	17 (60.7) ^a^	0.042	7 (63.6)	61 (76.3)	0.460	51 (70.8)	17 (60.7)	0.330
	No	4 (36.4) ^a,b^	8 (15.4) ^b^	11 (39.3) ^a^		4 (36.4)	19 (23.8)		21 (29.2)	11 (39.3)	
Postdrome	Yes	11 (100.0)	47 (90.4)	0 (0.0)	0.246	11 (100.0)	47 (82.5)	0.197	58 (92.1)	0 (0.0)	**< 0.001**
	No	0 (0.0)	5 (9.6)	5 (9.6)		0 (0.0)	10 (17.5)		5 (7.9)	5 (100.0)	

Note: Each superscript letter (a, b, c) indicates a genotypic subset in which the proportions within the column do not significantly differ from each other ( *p* > 0.05) in the Bonferroni adjustment.

The distribution of genotypes was consistent with the expected Hardy-Weinberg equilibrium ( *p* > 0.05) in both the migraine and control groups

## DISCUSSION

The main finding of the current study was the associations regarding the *IL1A* -889C > T variant and some clinical characteristics of migraine. The CT genotype was associated with a higher frequency of aura compared with the TT genotype, while the CT and TT genotypes were associated with a higher frequency of osmophobia compared with the CC genotype. Additionally, prodromal symptoms were more frequent in the patients carrying the CT genotype compared with those with the TT genotype.

The -889C > T variant is located in the regulatory region of the *IL1A* gene, where the substitution of cytosine with thymine might increase protein synthesis due to enhanced activity in the promoter region, leading to the emergence of a new transcription factor binding site, Skn-1. A previous study 19 showed that IL-1α cytokine production was higher in the TT genotype, followed by the CT genotype, and the lowest levels were observed in the CC genotype.

Aura is a transient neurological symptom that occurs immediately before or during a migraine attack. It arises from cortical spreading depression, a phenomenon involving neuronal and glial depolarization propagating throughout the cerebral cortex. This leads to the release of H ^+^ , K ^+^ , adenosine triphosphate (ATP), and glutamate, activating nociceptive meningeal nerve endings and adjacent immune cells. 20 A higher presence of aura diagnosis was associated with the CT genotype, as well as CC + CT genotypes in the codominant and recessive models, respectively ( Table 3 ). These genotypes produce low amounts of cytokine, 19 and this effect on the aura might be due to the reduced physiological effect of IL-1α, which is produced and expressed in the membrane of various cell types, including brain astrocytes, during homeostatic balance. 11 A small study by Boćkowski et al. 12 involving 21 children and adolescents found higher levels of IL-1α in migraine with aura. Additionally, Rainero et al. 13 (2002) analyzed the *IL1A* -889C > T variant in individuals with migraine, and those carrying the TT genotype were associated with a higher frequency of migraine with aura compared with carriers of other genotypes. These findings contrast with the results of the present study. However, Rainero et al. 13 did not analyze genetic variants, and the study by Boćkowski et al. 12 comprised only 21/152 (13.8%) individuals diagnosed with aura, whereas, in the current study, we identified 42/92 (45.7%) patients with aura. This difference in aura prevalence might be due to the use of the first edition of the headache classification 21 in these two aforementioned studies, while the present study employed the third edition. 17 The diverse methods used to diagnose aura may explain the discrepant findings among studies. Further research is needed to understand these conflicting results.

Osmophobia is the intolerance to odors that can occur before, during, and between migraine attacks. It is considered a specific symptom to diagnose migraine, 22 and its presence during the interictal period is associated with greater migraine-related disability. 23 However, the mechanism behind its occurrence has not been fully understood yet. A study evaluating the olfactory bulb in migraine patients compared with controls revealed a smaller olfactory bulb volume in the migraine group compared to the controls. Specifically, the left olfactory bulb was smaller in individuals with migraine and osmophobia compared with those with migraine but without osmophobia. 24 Unlike other brain areas, the microglia in the olfactory bulb continuously express toll-like receptor 2 (TLR-2), which mediates cytokine production. This suggests that the olfactory bulb may act as a sensor or modulator of neuroinflammation. 25 Therefore, the presence of osmophobia associated with the CT and TT genotypes and the fact that carriers of these two genotypes produce higher levels of cytokine compared with carriers of the CC genotype may reflect increased IL-1α production sensitizing the olfactory bulb, a region more susceptible to inflammatory changes.

The present study has some important limitations, especially the small sample size. Additionally, migraine patients using prophylactic medication were not excluded, which could impact the clinical manifestations of the disease. However, the present was a study with a comparable control group, and it adds information about the *IL1A* -889 C > T variant, which is still poorly studied in migraines. To our knowledge, this was the first study that associated osmophobia with an inflammatory mechanism. Moreover, it provided a counterpoint to the effect of the *IL1A* -889C > T variant on aura. Further studies are needed to confirm or refute these findings.
